# Supplementary material for: Are Antisense Long Non-Coding RNA Related to COVID-19?
Source: Biomedicines. 2022 Nov 1;10(11):2770. doi: 10.3390/biomedicines10112770 (PMC9687826; doi:10.3390/biomedicines10112770)
Supplement: Supplementary file 1 [file biomedicines-10-02770-s001.zip › biomedicines-1906920-supplementary.pdf]

Table (S1) primers list.

NCBI Reference Sequence	name	Forward primer	Reverse primer	Base pair
NM_014053.4	FLVCR1	CCCACAGACCAAGAACC	CCACCACATACAAACCC	156 bp
NR_027286.1	FLVCR1-DT	TGCTGATGTGCCCACTAAAG	CCATCATAGCCCGTCTCAGT	153 bp
NM_001355243.2	NCBP2AS2	TTTCCTCTGCAAGTGGGACT	CATGCCTATCAACCAGCTCA	154 bp
NR_102735.1	DBH-AS1	TTTGCCAGCATTGTTCTCTG	CGGACTCAGTTCCCTTTTGA	152 bp
NR_137424.1	A2M-AS1	GAACAGGACTCCAGCAAAGC	GGGCACTATGCTACCCAGAA	149 bp
NM_001101.5	Beta actin	GTGGATCAGCAAGCAGGAGT	AAAGCCATGCCAATCTCATC	147 bp

Table (S2): Univariate and multivariate Logistic regression analysis for the parameters affecting mortality

	Univariate		#Multivariate	
	**p**	**OR (95%C.I)**	**p**	**OR (95%C.I)**
**A2M-AS1**	**0.020***	0.001(0.0 – 0.350)	0.092	0.0(0.0 – 7.673)
**FLVCR1**	**0.267**	0.296(0.034 – 2.540)		
**DBH-AS1**	**0.011***	1.100(1.022 – 1.184)	0.120	1.278(0.938 – 1.742)
**FLVCR1-DT**	**0.009***	1.056(1.014 – 1.100)	0.117	1.217(0.952 – 1.556)
**NCBP2AS2-1**	**0.993**	–		
**Hemoglobin (gm/dl)**	**0.311**	1.351(0.755 – 2.417)		
**WBC (x103/ul)**	**0.950**	1.102(0.051 – 23.809)		
**Platelets**	**0.862**	1.015(0.861 – 1.196)		
**Lymphocytes (103/ul)**	**0.804**	0.869(0.288 – 2.623)		
**D.dimer (mg/ml)**	**0.263**	0.029(0.0 – 14.099)		
**CRP (mg/L)**	**1.000**	1.0(0.982 – 1.019)		
**Ferritin (ng/ml)**	**0.660**	1.022(0.928 – 1.125)		
**IL6 (pg/ml)**	**0.176**	0.503(0.186 – 1.360)		

OR: Odd`s ratio

C.I: Confidence interval LL: Lower limit UL: Upper Limit

#: All variables with p<0.05 was included in the multivariate

*: Statistically significant at $p \leq 0.05$

Table (S3): Univariate and multivariate Logistic regression analysis for the parameters affecting complications

	Univariate		#Multivariate	
	**p**	**OR (95%C.I)**	**p**	**OR (95%C.I)**
**A2M-AS1**	**0.162**	0.198(0.020 – 1.921)		
**FLVCR1**	**0.772**	0.955(0.698 – 1.305)		
**DBH-AS1**	**0.023***	1.063(1.009 – 1.120)	0.189	1.042(0.980 – 1.107)
**FLVCR1-DT**	**0.004***	1.046(1.014 – 1.079)	0.025*	1.039(1.005 – 1.074)
**NCBP2AS2-1**	**0.005***	1.109(1.032 – 1.192)	0.105	1.069(0.986 – 1.160)
**Hemoglobin (gm/dl)**	**0.809**	0.954(0.652 – 1.395)		
**WBC (x103/ul)**	**0.747**	0.710(0.089 – 5.681)		
**Platelets**	**0.301**	0.943(0.844 – 1.054)		
**Lymphocytes (103/ul)**	**0.698**	0.801(0.261 – 2.456)		
**D.dimer (mg/ml)**	**0.648**	0.393(0.007 – 21.595)		
**CRP (mg/L)**	**0.528**	1.004(0.991 – 1.017)		
**Ferritin (ng/ml)**	**0.502**	0.976(0.909 – 1.048)		
**IL6 (pg/ml)**	**0.825**	1.055(0.656 – 1.697)		

OR: Odd`s ratio
C.I: Confidence interval LL: Lower limit UL: Upper Limit
#: All variables with $p<0.05$ was included in the multivariate
*: Statistically significant at $p \leq 0.05$

Table (S4): **Univariate and multivariate Logistic regression analysis for the parameters affecting mechanical ventilation**

	Univariate		#Multivariate	
	**p**	**OR (95%C.I)**	**p**	**OR (95%C.I)**
**A2M-AS1**	**0.466**	0.377(0.027 – 5.179)		
**FLVCR1**	**0.261**	0.448(0.111 – 1.818)		
**DBH-AS1**	**0.005***	1.104(1.030 – 1.182)	0.094	1.089(0.986 – 1.202)
**FLVCR1-DT**	**0.004***	1.054(1.017 – 1.092)	0.049*	1.061(1.0 – 1.127)
**NCBP2AS2-1**	**0.002***	1.229(1.077 – 1.403)	0.034*	1.171(1.012 – 1.355)
**Hemoglobin (gm/dl)**	**0.476**	1.186(0.742 – 1.894)		
**WBC (x103/ul)**	**0.938**	1.109(0.084 – 14.632)		
**Platelets**	**0.283**	1.087(0.934 – 1.265)		
**Lymphocytes (103/ul)**	**0.712**	0.410(0.004 – 46.436)		
**D.dimer (mg/ml)**	**0.412**	0.126(0.001 – 17.868)		
**CRP (mg/L)**	**1.000**	1.0(0.984 – 1.016)		
**Ferritin (ng/ml)**	**0.639**	1.020(0.940 – 1.106)		
**IL6 (pg/ml)**	**0.169**	0.592(0.281 – 1.250)		

OR: Odd`s ratio
C.I: Confidence interval LL: Lower limit UL: Upper Limit
#: All variables with $p<0.05$ was included in the multivariate
*: Statistically significant at $p \leq 0.05$
